# From Shared Mechanisms to Precision Breeding: Engineering Cold and Drought Cross-Tolerance in Crops

**DOI:** 10.3390/ijms27052497

**Published:** 2026-03-09

**Authors:** Xue Yang, Zi-Chang Jia, Yan Liu, Xue Wang, Jia-Jia Chen, Ying-Gao Liu, Mo-Xian Chen

**Affiliations:** 1 State Key Laboratory of Green Pesticide, Center for R&D of Fine Chemicals, Guizhou University, Guiyang 550025, China; xueyang202001@163.com (X.Y.); jiazc973@163.com (Z.-C.J.); wx751484671@163.com (X.W.); chenjj220@163.com (J.-J.C.); 2 State Key Laboratory of Crop Biology, College of Life Science, Shandong Agricultural University, Tai’an 271018, China; 17866708180@163.com; 3 Co-Innovation Center for Sustainable Forestry in Southern China, College of Biology and the Environment, Nanjing Forestry University, Nanjing 210037, China

**Keywords:** cold stress, drought stress, cross-tolerance, signaling network, genome editing, molecular design breeding

## Abstract

Low temperature and drought are among the most pervasive abiotic stresses limiting crop productivity worldwide, and their frequent co-occurrence or alternation imposes compounded constraints on agricultural sustainability. Increasing evidence supports cross-tolerance, whereby exposure to one stress enhances resistance to another, as an emergent property of shared signaling networks and integrative regulatory layers. In this review, we summarize recent advances in understanding cold–drought cross-talk, from early stress perception and secondary messengers to hormonal coordination via abscisic acid, transcriptional reprogramming centered on dehydration responsive element binding protein/C repeat binding factor (DREB/CBF) modules, and longer-term regulatory memory mediated by chromatin remodeling and biomolecular condensates. Importantly, we further discuss how these mechanistic insights can be translated into precision breeding strategies, including genome editing, allele mining, and backcross-assisted introgression, to accelerate the development of crop varieties with stable multi-stress tolerance. Finally, we highlight future directions for integrating multi-omics, high-throughput phenotyping, and data-driven approaches to enable efficient molecular design breeding for complex stress environments.

## 1. Introduction

Environmental stress typically refers to factors triggered by environmental conditions that adversely affect plant survival, growth, and development (such as high temperatures, low temperatures, drought, salinity, waterlogging, heavy metal contamination, intense light, and mineral nutrient deficiencies) [1,2,3]. With global climate change, the frequency, intensity, and duration of environmental stresses are all increasing, becoming a primary factor limiting crop yield and quality while threatening global food security [4]. In many instances, plants face not a single stress but the combined effects of multiple stresses. When confronted with complex stress combinations, plants respond through the coordinated action of multiple signal transduction pathways [5,6,7,8]. Cold and drought are globally important abiotic stressors, and they often impose substantial limitations on crop productivity [4,9]. Low-temperature episodes in temperate and high-elevation regions can compromise stand establishment and subsequent vegetative and reproductive development [10,11]. Meanwhile, drought is increasingly emerging as a dominant contributor to yield fluctuations in arid and semi-arid agricultural systems [4,12]. Climate change will not only alter the frequency and intensity of these stress events, but also increase their unpredictability in timing and intensity [9]. Together, these shifts threaten global food security and challenge the sustainability of agricultural systems.

Under real-world farming environments, cold and drought typically co-occur or overlap rather than acting alone [13,14,15]. Moreover, chilling early in the season can modulate subsequent drought sensitivity when sensitive developmental processes are underway [13,16]. Compared with single-stress events, cold–drought sequences can impose amplified yield reductions, highlighting that understanding plant performance under variable, combined field stresses is essential [13,17,18]. Plant responses to abiotic stress are highly complex, arising from coordinated regulation that spans molecular, cellular, and whole-plant physiological levels [19,20]. When cold and drought co-occur or occur in sequence, their impacts are often non-additive, resulting in amplified injury in some contexts but improved tolerance in others [13,19]. The interaction is highly contingent on intensity and duration, when during development the stresses strike, and their order of arrival [20,21]. Physiologically, cold and drought share several core effects, including cellular dehydration, elevated oxidative stress, and disruption of membrane integrity [16,22,23,24]. Despite these shared impacts, plants typically activate distinct acclimation routes, including membrane lipid remodeling in response to cold and stomatal regulation when water becomes limiting [25,26]. This combination of overlapping injury and stress-specific adaptations makes breeding challenging, because gains in tolerance to one stress may come at the expense of performance under another [27,28]. Given the increasing variability and unpredictability of these events, evaluating crop performance under temporally combined cold–drought scenarios is increasingly necessary for reliable yield stability.

**Figure 1:**
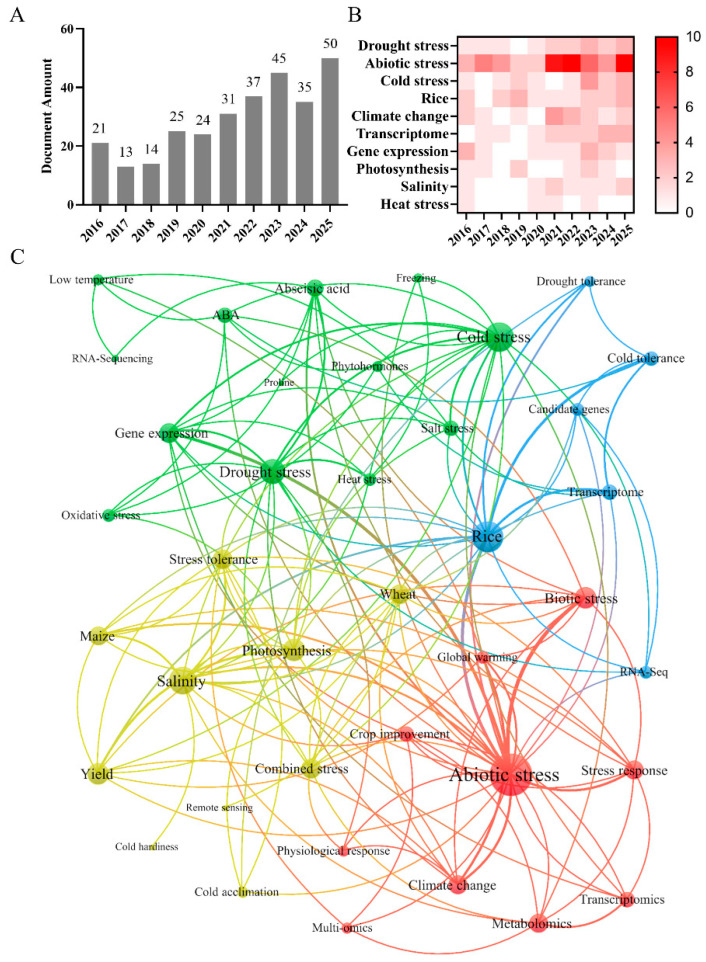
Bibliometric analysis of studies related to cold stress and drought stress based on the Web of Science Core Collection database from January 2016 to December 2025. (**A**) Histogram of literature statistics. The number of literature retrieved and counted by keywords. (**B**) Burst word detection analysis. Length represents the burst status duration; color saturation indicates citation burst strength. The timeframe was 2016–2025, with a threshold set to Top 10. (**C**) Keyword co-occurrence network. Nodes represent unique keywords; node size is proportional to the number of references; node colors indicate modules. Different colors represent distinct thematic clusters identified by VOSviewer: red (abiotic stress and omics), blue (rice stress tolerance and transcriptomics), green (stress signaling and gene expression), and yellow (crop physiology and yield). The search field was set to keywords, with the search term combination being “low-temperature stress OR drought stress OR combined stress”. The literature types were article and review, the search date was 3 January 2026, and the search scope was from January 2016 to December 2025. Following retrieval, duplicate documents were filtered out to yield the final set of valid literature for subsequent analysis. Correlation analyses were conducted using bibexcel (Version 2016-02-20), Pajek (Pajek64 Portable 6.01), VOSviewer (version 1.6.20), and GraphPad Prism 9 software.

In this review, we summarize recent advances in cold–drought stress research and explore how these mechanistic insights can be translated into precision breeding strategies. We highlight future research directions: integrating multi-omics technologies, high-throughput phenotyping, and data-driven approaches to achieve efficient molecular design breeding under complex stress conditions. Concurrently, we conduct an analysis of the existing literature on cold–drought combined stress within the Web of Science Core Collection database, shedding light on global research focal points and emerging trends through bibliometric analysis (refer to Figure 1 for details).

## 2. Independent and Overlapping Response Modules to Cold and Drought Stress

### 2.1. Similar Genes Are Induced by Dehydration and Cold Stress

Technology employing cDNAs or oligonucleotides has become a powerful tool for analyzing the gene expression profiles of plants exposed to abiotic stresses, such as cold and drought [29,30,31]. There are two predominant forms of microarray technology available, the cDNA microarray and the oligonucleotide microarray, which are used by GeneChip [32,33]. Both kinds of microarray technology are useful in identifying stress-inducible genes for the promoter sequences and stress-related transcription factors, opening a way to analyze gene-expression profiles in abiotic stress responses [34,35,36].

Recently, a variety of cold- and drought-inducible genes were identified using a cDNA microarray containing approximately 7000 independent *Arabidopsis* full-length clones [37]. Overlapping responses to osmotic and cold stress were accompanied by distinct changes in the expression of a suite of genes [38]. This suggests that similar biochemical processes function in cold- and dehydration-stress responses [39,40]. Genes induced in plants exposed to adverse stresses are thought to function not only in protecting cells by producing important metabolic proteins and cellular protectants, but also in regulating genes that are involved in transducing the stress response signaling pathway [41,42]. In *Arabidopsis*, these genes include responsive to dehydration (RD), early responsive to dehydration (ERD), cold-regulated (COR), low-temperature induced (LTI), and cold-inducible (KIN) [43,44,45,46]. The expression of RD and COR homologues in wheat and rice is also induced by low temperatures and drought, and their overexpression significantly enhances crop stress tolerance [13]. These stress-inducible genes suggest that the responses of plants to dehydration and cold are complex. Some of the stress-inducible genes are overexpressed in transgenic plants that have enhanced stress tolerance, suggesting that their gene products function in stress tolerance [47,48,49].

### 2.2. Regulons in Cold and Drought Stress-Responsive Gene Expression

Molecular studies in plants have shown that several genes with various functions are induced by cold and drought. For several stress-inducible genes, cis-acting elements in promoter regions and the corresponding transcription factors that affect the expression of these genes have been analyzed in *Arabidopsis*. In particular, understanding regulatory gene networks in drought and cold stress-inducible expression profiles depends on successful functional analyses of cis-acting elements.

The dehydration-responsive element (DRE)/C-repeat (CRT), a cis-acting element, is involved in osmotic stress- and cold stress-inducible gene expression. Transcription factors that bind to the DRE/CRT were isolated and named DRE-binding protein 1 (DREB1)/CRT-binding factor (CBF) and DREB2. DREB1A/CBF3, DREB1B/CBF1 and DREB1C/CBF2 regulons are involved in cold stress-responsive gene expression, whereas the DREB2 regulon is involved in osmotic stress-responsive gene expression [50,51]. For instance, in potatoes, the DREB1/CBF protein family has been demonstrated to be associated with heat and cold tolerance [52], whilst in maize, ZmDREB2A participates in drought responses [53]. This indicates that the cold/drought stress response mechanism mediated by DREB/CBF regulators exhibits extensive conservation across plants.

The interactions among different types of cis-acting elements function in cross-talk between different signals [54]. The cross-talk between cold and drought signal pathways could be explained by the presence of promoter elements in the stress-inducible genes. Many drought- and cold-inducible genes contain two *cis*-acting elements, namely a dehydration-responsive element (DRE; TACCGACAT) and an abscisic acid (ABA)-responsive element (ABRE; ACGTGG/TC) in their promoters [55,56,57]. Both the CRT and low-temperature-responsive element (LTRE) contain an A/GCCGAC motif that forms the core of the DRE sequence [58,59]. DRE/CRT functions cooperatively with ABRE as a coupling element in ABA-responsive gene expression in response to drought stress, which indicates that interactions between different transcription machineries function to provide cross-talk between different stress signaling pathways.

The RD29A/COR78/LTI78 gene is induced by drought, cold and ABA [60]. However, this gene is induced in *aba* or *abi* mutants by both drought and cold stresses, which indicates that it is governed by both ABA-dependent and ABA-independent regulation under drought and cold conditions. The promoter of the RD29A/COR78/LTI78 gene has been found to contain two major cis-acting elements, the ABRE and the DRE/CRT, that are involved in stress-inducible gene expression [61].

Various transcription factors interact with cis-acting elements in promoter regions and form a transcriptional initiation complex. The transcriptional initiation complex then activates RNA polymerase to start transcription of stress-responsive genes from the TATA box upstream of the transcriptional initiation sites. In this process, various interactions between transcription factors and cis-acting elements function as molecular switches to determine transcription initiation events.

The RD29A/COR78/LTI78 cDNAs encoding DRE-binding proteins C-repeat-binding factor 1(CBF1), DRE-binding protein1A (DREB1A), and DREB2A have been isolated by the yeast one-hybrid screening method [60,62]. These proteins specifically bind to the DRE/CRT sequence and activate the transcription of genes driven by the DRE/CRT sequence in *Arabidopsis*.

The homolog of DREB2A was isolated and identical to DREB2B [63]. Two homologs of CBF1/DREB1B, CBF2 and CBF3, are identical to DREB1C and DREB1A [64]. Expression of the DREB1/CBF genes is induced by cold stress and expression of the DREB2 genes is induced by drought stresses [63]. Both DREB1/CBF and DREB2 proteins bind to DRE, but DREB1/CBFs are thought to function in cold-responsive gene expression, whereas DREB2s are involved in drought-responsive gene expression, which indicates that cross-talk between drought- and cold-responsive gene expression occurs on a cis-acting element DRE.

### 2.3. ABA in Cold and Drought Stress Response

Drought and cold stresses cause an increased accumulation of ABA, which plays important roles in adaptive stress responses to environmental stimuli in plants [65,66,67,68].

The ABA core signal pathway is composed of pyrabactin resistance 1-like/protein phosphatase 2C/sucrose non-fermenting 1-related protein kinase 2 (PYL/PP2Cs/SnRK2s). The model of the ABA signal is interaction between PYL and PP2C, which represses PP2C activity, resulting in SnRK2s activated phosphorylation to trigger the plant stress response.

Many drought- and cold-stress-inducible genes are induced by exogenous ABA treatment. These genes contain potential ABA-responsive elements (ABREs, PyACGTGGC) in their promoter regions [69].

Genetic analysis of the ABA-deficient mutants los5/aba3 and los6/aba1 of *Arabidopsis* showed that ABA plays a pivotal role in osmotic stress-regulated gene expression [70,71]. Perception and signaling factors such as PYL4 can also be used to improve stress tolerance [72]. This application has also been validated in crops such as rice [73], providing important molecular targets for crop stress-tolerant breeding. The rd29A promoter contains not only DRE but also an ABRE. An ABRE cis-acting element and basic leucine zipper (bZIP) transcription factors function in ABA-responsive gene expression. The rd29A gene is therefore controlled by three independent regulatory systems. These results indicate that complex molecular responses to various environmental stresses may be mediated by both complex regulatory systems of gene expression and signal transduction, and by cross-talk between these systems.

## 3. Core Integrative Networks Underlying Cross-Tolerance

### 3.1. Rapid Perception and Signal Initiation

Plants not only have stress-specific signal transduction pathways such as the ICE-CBF-COR signaling pathway of cold stress but they also cross-talk between abiotic stresses, for instance the ABA signaling [74]. Genetic and molecular analyses have suggested that there is extensive cross-talk between osmotic stress, temperature stress, and ABA responses. Expression of 40% of drought-inducible genes was also induced by cold stress, which indicates a relationship between pathways in response to cold versus drought stress [42].

The cross-talk between cold and drought pathways could be explained by the presence of promoter elements in the stress-inducible genes [37]. Many drought- and cold-inducible genes contain both DRE/CRT and ABRE motifs in their promoters. DRE/CRT functions cooperatively with ABRE as a coupling element in ABA-responsive gene expression in response to drought stress, which indicates that interactions between different transcription machineries function to provide cross-talk between different stress signaling pathways [50,75]. The molecular mechanisms regulating gene expression in response to dehydration and cold stresses have been studied by analyzing the cis- and trans-acting elements that function in ABA-independent and ABA-responsive gene expression during the stresses in *Arabidopsis.*

Cloning and transgenic analysis of a DREB1-related transcription factor, CBF4 in *Arabidopsis*, showed that regulation of DREs may also be mediated by an ABA-dependent pathway [76,77].

Genes of the CBF/DREB1 family are mainly induced by cold stress, but the drought-inducible gene CBF4 functions to provide cross-talk between DREB2 and CBF/DREB1 regulatory systems. Overexpression of CBF4 in *Arabidopsis* resulted in constitutive expression of CRT/DRE containing stress-responsive genes and enhanced tolerance to drought and freezing stresses [76]. Overexpression of the CBF4 gene in barley also enhances the plant’s tolerance to stress [78].

### 3.2. Transcriptional Reprogramming

The mutant screening system using transgenic *Arabidopsis* plants containing a stress-inducible promoter: Luciferase reporter gene (LUC) provides a powerful tool for the genetic analysis of cold and drought stress signal transduction pathways [79]. To facilitate genetic screens for stress-signaling mutants, transgenic *Arabidopsis* were engineered to express the firefly LUC under control of the RD29A promoter, which contains both ABRE and DRE/CRT.

Many *Arabidopsis* mutants, such as los5/aba3 [70], los2 [80], los6/aba1 [71], have been isolated and have altered the induction of stress-responsive genes using this system. Compared with wild-type RD29A-LUC plants, these mutants exhibited altered expression of the RD29A::LUC gene at a constitutive (cos), high (hos), or low (los) level in response to various abiotic-stress or ABA treatments [79]. The occurrence of mutations with differential responses to drought, cold, high-salinity and/or ABA treatments predicts cross-talk among the drought, salinity, cold and ABA signal transduction pathways [81]. Transcription of the RD29A gene in response to osmotic and cold stresses is mediated by both ABA-dependent and ABA-independent pathways [75].

**Figure 2:**
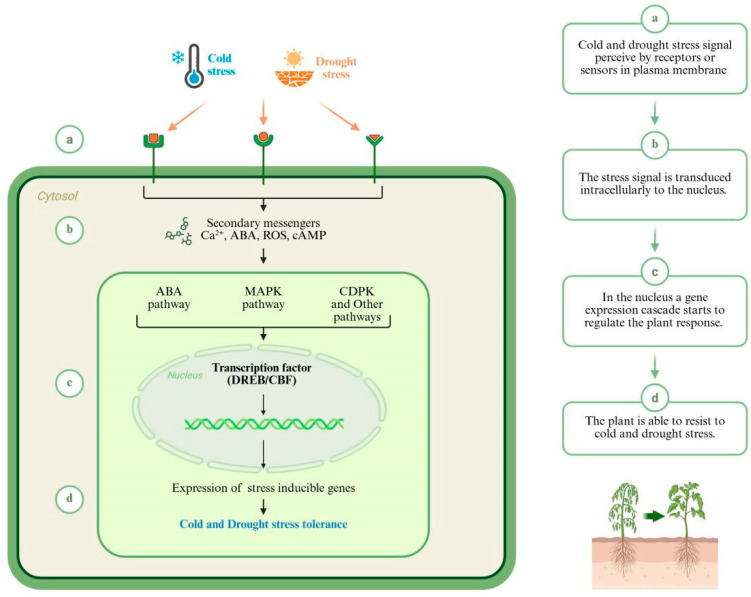
The core shared signaling network in cold–drought cross-tolerance. (a), Cold and drought stress are perceived by receptors at the plasma membrane, triggering an influx of calcium ions (Ca^2+^) and a burst of reactive oxygen species (ROS). (b) These secondary messengers activate downstream kinase cascades (e.g., MAPK) and promote the biosynthesis and signaling of abscisic acid (ABA). (c), The Ca^2+^, ROS, and ABA signals converge on key transcription factor families, most notably the DREB/CBF proteins, which act as central hubs for signal integration. (d) These master regulators bind to cis-elements (e.g., DRE/CRT) in the promoters of a large suite of downstream target genes, leading to the concerted activation of protective mechanisms. These include the synthesis of osmoprotectants, late embryogenesis abundant (LEA) proteins, and antioxidant enzymes, which collectively enhance cellular resilience to both stresses. This shared signaling network provides the molecular basis for cross-tolerance and represents a prime target for synthetic biology approaches aimed at engineering multi-stress-resistant crops.

Under cold and drought stress conditions, stress-induced condensates have been demonstrated to promote rapid and efficient transcriptional activation [82,83]. In cold conditions, Frigida (FRI) and Frigida-like (FRL) form biomolecular condensates, preventing FRI from localizing to the FLC transcriptional activation site to promote transcription [83]. Under drought conditions, SEUSS condensation enhances *Arabidopsis* tolerance to hyperosmotic stress [82]. In cross-tolerance, these condensates may function as molecular accelerators, amplifying the activation rate and intensity of pre-activated genes during repeated or continuous stress exposure. For instance, during cold induction, FRI undergoes reversible liquid–liquid phase transition (LLPS) to form nuclear condensates, dissociating FRI from FLC transcriptional activation. Upon temperature elevation, FRI nuclear condensates revert to a dispersed state, enabling FRI to bind the FLC promoter and promote expression [83,84,85]. By linking epigenetic pre-activation with transcriptional sub-spatial clustering, LLPS establishes a mechanistic bridge between stress memory and rapid gene induction [86]. Under cold stress in rice, the OsSRO1c-OsDREB2B complex undergoes a protein phase transition, enhancing the plant’s cold tolerance [87]. This further substantiates the role of condensates in the cross-tolerance of cold and drought stress in crops. The core signaling network for cold–drought cross-tolerance is detailed in Figure 2. The core molecular components mediating cross-tolerance to low temperature and drought in plants, along with their potential applications, are summarized in Table 1.

## 4. Breeding Technologies for Cold–Drought Cross-Tolerance

### 4.1. Network-Derived Molecular Targets

Understanding the mechanisms underlying cold–drought cross-tolerance provides a rational basis for crop improvement, as shared regulatory hubs often represent more effective targets than stress-specific endpoints. Notably, integrative nodes that link early signaling (Ca^2+^ and ROS) (Figure 2) [16,101], hormonal coordination via ABA, and transcriptional control (for example, DREB/CBF-centered modules) [13,102] can shape wide-ranging protective responses, ranging from osmotic adjustment to antioxidative defenses and cellular stabilization.

Targeting regulatory hubs must be approached with caution, as increases in stress tolerance can be accompanied by growth penalties when regulatory amplitude or timing is mistuned [103,104]. In a breeding context, the most promising targets are thus those that optimize, rather than maximize, stress responses to balance resilience with productivity. Target prioritization should incorporate effect size under realistic cold–drought sequences, pleiotropic impacts on development and yield components, and stability across environments and genetic backgrounds [100,105]. This target-first perspective provides a coherent framework for integrating discovery with application, allowing defined hubs to be advanced through genome editing, allele mining, and introgression in parallel.

Based on current evidence, core candidate target classes include transcription factor hubs (such as DREB/CBF and their interacting regulators) [92], components of ABA signaling (receptors and downstream kinases/phosphatases) [106], ROS production and scavenging modules [107], and regulatory memory factors [108], including chromatin regulators and condensate-forming proteins. For each class, modern breeding strategies can leverage either naturally occurring functional alleles in germplasm or engineered alleles generated through genome editing, followed by validation under field-relevant stress regimes [100].

### 4.2. Genome Editing for Precision Improvement of Stress Tolerance

Genome editing, especially CRISPR/Cas-based approaches, provides a powerful route to translate cross-tolerance mechanisms into actionable alleles [100,109]. Unlike transgenic strategies relying on foreign gene overexpression, editing can modify endogenous loci in ways that mimic or accelerate natural evolution. In the context of cold–drought cross-tolerance, three editing modes are particularly relevant: (i) loss-of-function edits in negative regulators to enhance pathway sensitivity [110], (ii) promoter or cis-regulatory editing to fine-tune expression timing and amplitude [111], and (iii) multiplex editing to adjust multiple network nodes simultaneously [112,113].

Many stress pathways are constrained by negative regulatory components (for example, phosphatases that suppress kinase signaling) [114]. Targeted disruption or weakening of such negative regulators can enhance stress responsiveness, but the risk of constitutive stress signaling and yield penalties must be managed [110]. Practical breeding-oriented editing therefore favors partial or conditional modulation—such as editing specific domains, isoforms, or regulatory elements—over complete loss-of-function when pleiotropy is expected [115,116].

For hub transcription factors and ABA-related regulators, promoter editing offers a route to “expression tuning” without altering coding sequences [111,117]. By modifying cis-elements associated with stress inducibility or tissue specificity, it is possible to strengthen responses under stress while minimizing unnecessary expression in favorable conditions [118]. This approach is especially attractive for traits with known growth–stress trade-offs, because it aims to optimize rather than maximize stress outputs [119].

Cross-tolerance is rarely governed by a single locus. Multiplex editing can create allele combinations that coordinate upstream signaling, transcriptional control, and downstream protection [21,120]. Still, network-level edits require careful phenotyping under realistic stress regimes, because some combinations may generate antagonistic interactions or over-activation [121].

### 4.3. Allele Mining and Haplotype Optimization

Natural genetic variation remains an indispensable resource for improving complex stress tolerance. Many crops and their wild relatives have evolved under strong climatic constraints, accumulating alleles that modulate shared stress hubs while maintaining growth and reproduction [122,123,124]. Allele mining aims to identify such favorable variants and to link them to phenotypic performance under combined or sequential stress conditions [125,126]. A major advantage of allele mining is that naturally occurring variants often embody pre-optimized trade-offs, shaped by selection in fluctuating environments [127]. Instead of producing large perturbations, natural alleles may subtly adjust pathway sensitivity, expression kinetics, or tissue specificity—features that align well with the breeding goal of stable performance rather than maximal stress response. Therefore, focusing on haplotypes around key hubs can be more informative than single polymorphisms [87,128].

In practice, allele mining benefits from integration across multiple data types: stress-responsive transcriptomes to prioritize candidate regulators, population genomics to detect selection signals, and high-throughput phenotyping to quantify performance under controlled and field-like stress patterns [129]. Importantly, cold–drought cross-tolerance should be assessed using stress sequences and recovery phases, as tolerance phenotypes can differ substantially between simultaneous stress and stress priming scenarios. Haplotype optimization also provides a bridge between mining and editing [130]. Once beneficial haplotypes are identified, genome editing can be used to recreate or refine key variants in elite backgrounds, and marker-assisted strategies can speed up introgression of naturally favorable haplotypes [128,131].

### 4.4. Backcross-Assisted Introgression and Trait Pyramiding

While genome editing accelerates the creation of targeted alleles, backcross-assisted introgression remains a practical and widely applicable route to move stress-tolerance loci into elite cultivars [132,133]. Marker-assisted backcrossing enables breeders to introgress beneficial alleles from donor lines while minimizing linkage drag, which is particularly important when donors include wild relatives or landraces with undesirable agronomic traits [134,135]. For example, drought- and cold-adaptive alleles derived from wild barley or teosinte-related germplasm have been successfully transferred into elite cereal backgrounds using Marker-Assisted Backcrossing (MABC), with background selection accelerating recovery of yield and plant architecture while retaining stress resilience [136].

For cold–drought cross-tolerance, introgression strategies should ideally prioritize loci that act as integrative nodes or that confer robust downstream protection with minimal penalty [16]. Plant tolerance to combined stresses involves multiple pathways. In some instances, introgressed lines carrying multiple resistance loci may exhibit greater stability and superior performance under combined stresses compared to single-locus lines. However, simply combining multiple resistance loci does not always yield additive effects; certain combinations may produce antagonistic interactions detrimental to crop growth or yield [137].

A breeding-oriented framework typically includes: (i) validating donor alleles under sequential stress regimes [138], (ii) identifying diagnostic markers for rapid selection [132], (iii) backcrossing into elite backgrounds while monitoring key agronomic traits [139], and (iv) combining complementary loci through pyramiding [140]. Practical programs often add an early “background-sensitivity” screen, testing introgressed lines across two or three elite genetic backgrounds to detect context-dependent effects before large-scale deployment [141]. Importantly, the breeding value of a locus should be judged across environments, as cross-tolerance can be strongly influenced by climate patterns and management practices [139]. Only loci that repeatedly contribute to yield stability—rather than isolated stress survival—in multi-location trials should be advanced toward release.

### 4.5. Molecular Design Breeding

Molecular design breeding seeks to translate mechanistic, network-level understanding into actionable breeding decisions, thereby improving complex traits more efficiently [16]. Using cold–drought cross-tolerance as an example, molecular design breeding first needs to convert a network view into quantifiable objectives [114]. At the perception and signaling layers, it aims to moderately dampen excessive sensitivity (e.g., by fine-tuning the ABA receptor–PP2C–SnRK2 module and adjusting the activation thresholds of Ca^2+^ channels/OSCA osmosensors) [142,143]. At the shared downstream output layer, it strengthens common protective programs (e.g., increasing proline and soluble sugar accumulation to stabilize osmotic homeostasis; enhancing antioxidant capacity such as SOD/CAT/APX systems to maintain redox balance; reinforcing LEA/dehydrin function and membrane-lipid remodeling to protect membrane integrity) [144,145]. At the dynamic layer, it accelerates response kinetics (e.g., leveraging rapid activation of transcription-factor networks including CBF/DREB [146], NAC, and bZIP, improving the speed of re-stress responses through heat-shock proteins/HSP pathways, HSFA2-associated routes, and epigenetic priming/memory) [147,148,149]. Crucially, these improvements should be achieved without collapsing growth and yield stability (e.g., using tissue-specific or weakened promoters, or promoter editing to avoid whole-plant constitutive overexpression that can cause dwarfing, delayed maturation, or impaired grain filling) [117,150].

A pragmatic way to implement molecular design breeding is as a repeating cycle. Prioritize network nodes and alleles based on cross-tolerance network ranking (e.g., prioritize hubs that function in both cold and drought responses and have historically shown limited pleiotropic penalties, such as NCED/ABA biosynthesis, PP2C negative regulation [151,152,153], CBF/DREB transcriptional control [154], P5CS-mediated proline biosynthesis [155], TPS/TPP trehalose pathways [156,157], NHX-related ion/osmotic homeostasis [158], and APX-mediated antioxidation) [159,160,161]. Create or discover variation via gene editing or allele mining (e.g., using CRISPR to dial down a transcription factor by promoter tuning to achieve a dosage-dependent effect; or mining landraces and wild relatives for alleles that confer stronger root system architecture or more stable cold germination). Introgress and stack favorable alleles into elite backgrounds through backcrossing combined with marker-assisted selection and/or genomic selection (e.g., packaging loci controlling root angle/deep rooting together with loci associated with cold germination/low-temperature fertility into marker sets for generational tracking while avoiding linked deleterious effects) [162]. Evaluate under field-relevant temporal sequences and compound stress windows, capturing genuine stress dynamics (e.g., sequential stresses such as early spring cold shocks, recovery, drought during the jointing/heading stage, or combinations on diurnal scales like nocturnal cold with daytime high transpiration drought) [163]. Early-stage testing should include both single-stress treatments and seasonally realistic sequence–recovery regimes, because cross-tolerance is fundamentally an environment-dependent phenotype: the same genotype can show opposite outcomes under “cold-to-drought” versus “drought-to-cold” trajectories.

### 4.6. Case Studies: Successful Deployment of Cross-Tolerant Crops

**Figure 3:**
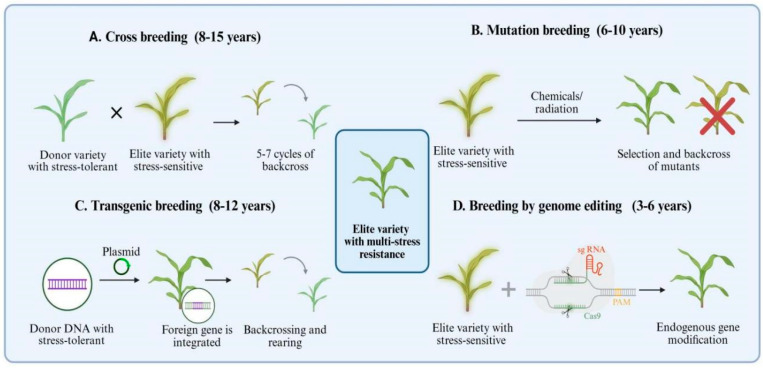
Timeline comparison of breeding strategies for developing crops with cold–drought cross-tolerance. (**A**) Cross breeding: Pyramiding beneficial alleles through multiple generations of crossing and backcrossing, resulting in the longest cycle with potential linkage drag. (**B**) Mutation breeding: Creating random variations via physical/chemical mutagenesis followed by screening, which shortens the cycle but remains highly stochastic. (**C**) Transgenic breeding: Introducing specific foreign genes to enhance stress tolerance, yet requiring extensive backcrossing and rigorous regulatory approval. (**D**) Gene editing breeding: Utilizing tools like CRISPR for precise, targeted modification of endogenous genes (e.g., key regulatory factors), enabling the most rapid assembly of desired traits and a significantly shortened breeding timeline.

Successful deployment of cold–drought cross-tolerant cultivars typically follows one of two routes. The first involves conventional breeding enhanced by marker-assisted selection and gene introgression, utilizing hybrid breeding and mutation breeding to progressively accumulate favorable alleles (Figure 3A,B). This pathway demonstrates significant advantages due to its broad applicability and adaptability across diverse cropping systems, though it may be constrained by the extended breeding cycle and the complexity of constructing trait architecture [164]. For instance, in wheat and barley, repeated selection combining frost resistance markers (such as loci associated with the CBF pathway) [146,165] with drought resistance trait markers (such as root depth or osmotic regulation capacity) [166] has yielded varieties exhibiting greater stability under continental climates, despite each breeding cycle spanning multiple years and yielding limited gains per cycle. Similarly, in rice cultivated in temperate or high-altitude regions, the progressive introduction of cold-tolerant alleles into japonica cultivars has enhanced seedling establishment under cool, intermittently dry conditions, though often requiring extensive backcrossing to restore yield and grain quality [167].

The second route relies on targeted creation or optimization of alleles through genome editing, followed by introgression into elite backgrounds and multi-environment evaluation (Figure 3C,D) [100,139]. This approach can substantially shorten the time needed to validate causal loci and to assemble allele combinations, but it remains sensitive to background effects and requires careful selection of targets that preserve yield-related traits [168]. For instance, editing regulatory regions of stress-responsive transcription factors or hormone signaling genes (rather than complete knockouts) has been explored in maize and rice to fine-tune cold and drought responses while minimizing penalties to growth [168]. In some cases, multiplex editing of several small-effect loci has produced lines with improved tolerance to combined stress in managed trials, yet performance varied once those alleles were placed into different elite genetic backgrounds. Across both routes, the most convincing success evidence comes from multi-location field trials demonstrating yield stability and agronomic performance under variable seasons that include cold and drought episodes [111,169]. A representative example is regional trial networks where candidate cultivars are tested across years that differ in sowing temperature, early-season frost risk, and late-season water availability, revealing that lines with similar single-stress tolerance can diverge markedly in yield stability under compound stress scenarios. Such trials often show that genotypes optimized for stress signaling balance and recovery capacity outperform those relying on extreme constitutive tolerance [139].

## 5. Conclusions and Future Prospects

Cold and drought, as common abiotic stresses, exert severe impacts on crop yields. In certain circumstances, these two stresses may co-occur as combined stress, further compromising the stability of crop production. Numerous studies have confirmed that plant responses to cold and drought exhibit significant overlap in early signal transduction, transcription, and translation pathways [16]. Consequently, gaining a deeper understanding of plant response mechanisms to combined stress holds significant importance for the optimization and improvement of crops. In practical breeding work, the identification and application of key regulatory nodes and alleles under complex stress conditions are of paramount importance. This will significantly accelerate the development of more stress-tolerant varieties and enhance the stability of grain yields.

Currently, research concerning plant tolerance to cold–drought stress has achieved considerable progress [170,171,172]. However, significant limitations remain in its practical application and the stable cultivation of crops exhibiting cross-tolerance to cold and drought.

In post-transcriptional regulation, mechanistic studies remain relatively scarce. Non-coding RNAs, alternative splicing, RNA stability, and translational control all play pivotal roles in stress responses [171]. Deepening our understanding of these mechanisms and translating them into practical agricultural applications is now a key focus. Here, multi-omics analysis and molecular validation can systematically identify key regulatory factors for crop improvement.

The environment crops encounter under combined stress conditions is complex, and single-analysis approaches alone cannot fully elucidate their regulatory networks. Consequently, it is often necessary to integrate data from genomics, epigenomics, transcriptomics, proteomics, and phenomics to determine the relationships between genes and phenotypes [173]. Moreover, altering a single component often proves insufficient to maintain crop stability across multiple stress environments. Consequently, constructing allele combinations that simultaneously enhance stress resistance while preserving growth and yield represents our core challenge. Future research should intensify efforts to uncover key regulatory networks and identify superior allele combinations. Only then can cold–drought cross-tolerance evolve from a descriptive concept into a tangible breeding objective.

## Figures and Tables

**Table 1:** Core molecular components mediating plant cross-tolerance between cold and drought stress and their potential synthetic biology applications.

Component		Role in Cross-Tolerance	Synthetic Biology Approach
DREB/CBF TFs	MhDREB2A [88], GmDREB [89], TaDREB2/3 [90], EgDREB1 [91]	Master regulators of cold- and drought-induced genes [92].	Engineered promoters & synthetic transcription factors.
ABA Signaling	PYLs-PP2Cs-SnRK2s-ABF2 [93], NRT1.1B [94], SnRK1 [95]	Core hub for stress signal transduction and amplification [96].	Orthogonal receptors for inducible control [93].
ROS Signaling	RBOHD [97]	Second messenger linking cold and drought signaling [97].	Compartmentalized scavenging modules [98].
Epigenetic Regulators	HDA6 [99]	Encode long-term stress memory via chromatin marks	Targeted editing (e.g., CRISPR-dCas9) [100].
Phase-Separating Proteins	FRI, SEUSS [82]	Form condensates for rapid, precise response [86].	Engineered proteins with synthetic IDRs.

## Data Availability

No new data were created or analyzed in this study. Data sharing is not applicable to this article.
